# Joint Modeling of Repeated Measurements of Different Biomarkers Predicts Mortality in COVID-19 Patients in the Intensive Care Unit

**DOI:** 10.1177/11772719221112370

**Published:** 2022-07-14

**Authors:** Kirby Tong-Minh, Yuri van der Does, Joost van Rosmalen, Christian Ramakers, Diederik Gommers, Eric van Gorp, Dimitris Rizopoulos, Henrik Endeman

**Affiliations:** 1 Department of Emergency Medicine, Erasmus University Medical Center, Rotterdam, The Netherlands; 2 Department of Biostatistics, Erasmus University Medical Center, Rotterdam, The Netherlands; 3 Department of Epidemiology, Erasmus University Medical Center, Rotterdam, The Netherlands; 4 Department of Clinical Chemistry, Erasmus University Medical Center, Rotterdam, The Netherlands; 5 Department of Intensive Care, Erasmus University Medical Center, Rotterdam, The Netherlands; 6 Department of Internal Medicine, Erasmus University Medical Center, Rotterdam, The Netherlands; 7 Department of Viroscience, Erasmus University Medical Center, Rotterdam, The Netherlands

**Keywords:** COVID-19, repeated measurements, join models, prediction model, intensive care unit, biomarkers

## Abstract

**Introduction:**

Predicting disease severity is important for treatment decisions in patients with COVID-19 in the intensive care unit (ICU). Different biomarkers have been investigated in COVID-19 as predictor of mortality, including C-reactive protein (CRP), procalcitonin (PCT), interleukin-6 (IL-6), and soluble urokinase-type plasminogen activator receptor (suPAR). Using repeated measurements in a prediction model may result in a more accurate risk prediction than the use of single point measurements. The goal of this study is to investigate the predictive value of trends in repeated measurements of CRP, PCT, IL-6, and suPAR on mortality in patients admitted to the ICU with COVID-19.

**Methods:**

This was a retrospective single center cohort study. Patients were included if they tested positive for SARS-CoV-2 by PCR test and if IL-6, PCT, suPAR was measured during any of the ICU admission days. There were no exclusion criteria for this study. We used joint models to predict ICU-mortality. This analysis was done using the framework of joint models for longitudinal and survival data. The reported hazard ratios express the relative change in the risk of death resulting from a doubling or 20% increase of the biomarker’s value in a day compared to no change in the same period.

**Results:**

A total of 107 patients were included, of which 26 died during ICU admission. Adjusted for sex and age, a doubling in the next day in either levels of PCT, IL-6, and suPAR were significantly predictive of in-hospital mortality with HRs of 1.523 (1.012-6.540), 75.25 (1.116-6247), and 24.45 (1.696-1057) respectively. With a 20% increase in biomarker value in a subsequent day, the HR of PCT, IL-6, and suPAR were 1.117 (1.03-1.639), 3.116 (1.029-9.963), and 2.319 (1.149-6.243) respectively.

**Conclusion:**

Joint models for the analysis of repeated measurements of PCT, suPAR, and IL-6 are a useful method for predicting mortality in COVID-19 patients in the ICU. Patients with an increasing trend of biomarker levels in consecutive days are at increased risk for mortality.

## Introduction

Coronavirus disease (COVID-19) caused by the novel Coronavirus (SARS-CoV-2) was declared a pandemic on the 11th of March 2020 by the World Health Organization.^1^ Approximately a third of the patients with COVID-19 require treatment at an intensive care unit (ICU) when they develop acute respiratory distress syndrome (ARDS).^2,3^ To manage hospital capacities, while providing the best care possible for as many patients, patient triage and information of prognosis of individual patients is required. Predicting disease severity is important for treatment decisions, especially when ICU capacity is limited by the overwhelming amount of admissions.^4^

Multiple predictors of mortality in COVID-19 patient have been studied since the start of the pandemic.^5^ These vary from routinely measured vital parameters and laboratory tests, demographic data to experimental biomarkers. Different biomarkers have been investigated in COVID-19, including C-reactive protein (CRP), procalcitonin (PCT), interleukin-6 (IL-6), and soluble urokinase-type plasminogen activator receptor (suPAR).^6-9^ These biomarkers are involved in different inflammatory pathways and are elevated in different kind of infections and have also been incorporated in different prediction models of disease severity or mortality.^10,11^

The majority of the previously studied prediction models are developed and validated using single measurements (cross sectional), even though many parameters are measured daily in ICU patients. When biomarkers levels rise or fall over time, this data can be used to predict disease progression and ultimately mortality.^12^ However, these changed over time in biomarkers are rarely studied in prognostic studies. Using repeated measurements in a prediction model may result in a more accurate risk prediction than the use of single point measurements.^13^

The goal of this study is to investigate the predictive value of repeated measurements of different biomarkers on mortality in patients admitted to the ICU with COVID-19.

## Methods

This study was a retrospective single center cohort study. We included patients admitted to the ICU of Erasmus University Medical Center, in Rotterdam, the Netherlands, with a confirmed COVID-19 infection between 1 March 2020 and 30 April 2020. Erasmus University Medical Center had an ICU capacity of 72 beds during the COVID-19 pandemic. The institutional review board waived informed consent for the retrospective use of clinical data of COVID-19 patients under protocol number MEC-2020 to 0381.

## Inclusion and Exclusion Criteria

Patients were included if they tested positive for SARS-CoV-2 by PCR test and if IL-6, PCT, suPAR were measured during any of the ICU admission days. There were no exclusion criteria for this study.

## Data Collection

Patient data including demographics, body mass index, and comorbidities were collected from the day of admission to the ICU. Biomarker data was recorded from every day as long as the patient was admitted to the ICU. Patients were followed up until discharge from the ICU or in-hospital death.

## Primary Outcome

The primary outcome of this study was ICU-mortality.

## Biomarker Measurements

In every patient, blood was drawn daily at 06.00 AM for laboratory testing. PCT was measured using E801 Elecsys BRAHMS PCT reagent and IL-6 was measured using E801 Elecsys IL-6 reagent, both on a COBAS 8000 (Roche Diagnostics, Switzerland). SuPAR was measured using a turbidimetric assay (Virogates, Denmark) on a COBAS 6000 (Roche Diagnostics, Switzerland). The values of these biomarkers were reported in the electronic patient records and available to the treating physician in the ICU.

## Sample Size Calculation

For this study we used a convenience sample of the patients admitted to the ICU in which additional biomarkers were measured. This period lasted from March to April 2020.

## Statistical Analysis

Normally distributed variables were reported as mean with standard deviation (SD), non-normally distributed variables as median with interquartile range (IQR). Differences in dichotomous variables between the survivors and the non-survivors were analyzed with chi-square tests. Differences in continuous variables were analyzed using an independent samples t-test for normally distributed data and a Mann-Whitney *U* test for non-normally distributed data.

For the baseline predictors age, sex, and body mass index (BMI) we presented standard Cox regression model analysis and Kaplan-Meier curves for the survival function.

Following, we continued in the analysis of the longitudinally measured biomarkers. This analysis was done using the framework of joint models for longitudinal and survival data. These models combine a linear mixed-effects model per biomarker that describes the patient-specific longitudinal trajectories. These estimated trajectories are then put in a Cox model for the time-to-death, also corrected for age and sex. Many of the biomarkers have limits of detection (either from above or below), and skewed distributions. To accommodate for these features, we used linear mixed models that account for censoring, and we transformed the biomarkers’ values using the logarithmic transformation with base 2. This means that the reported hazard ratios (HRs) express the relative change in the risk of death resulting from a doubling of the biomarker’s value in a day compared to no change in the same period. Due to a limited detection limit of suPAR and IL-6, we also calculated the HR for mortality when biomarkers increased by 20% in the next day. We used splines in the fixed and random effects parts for biomarkers with nonlinear shapes of the patient-specific longitudinal trajectories.

Statistical analyses were performed using “R” version 4.00.5. For joint modeling the package JMbayes2 version 0.1 to 6 was used.

## Results

Between 1st of March 2020 and 30th of April 2020, a total of 110 patients were admitted to the ICU with a confirmed COVID-19 infection. PCT, IL-6, or suPAR were measured in 107 of these patients. These 107 patients were included in the final analysis. In total, 26 patients died during ICU admission. There was missing data in BMI in 1 patient (0.9%).

**Figure 1.**
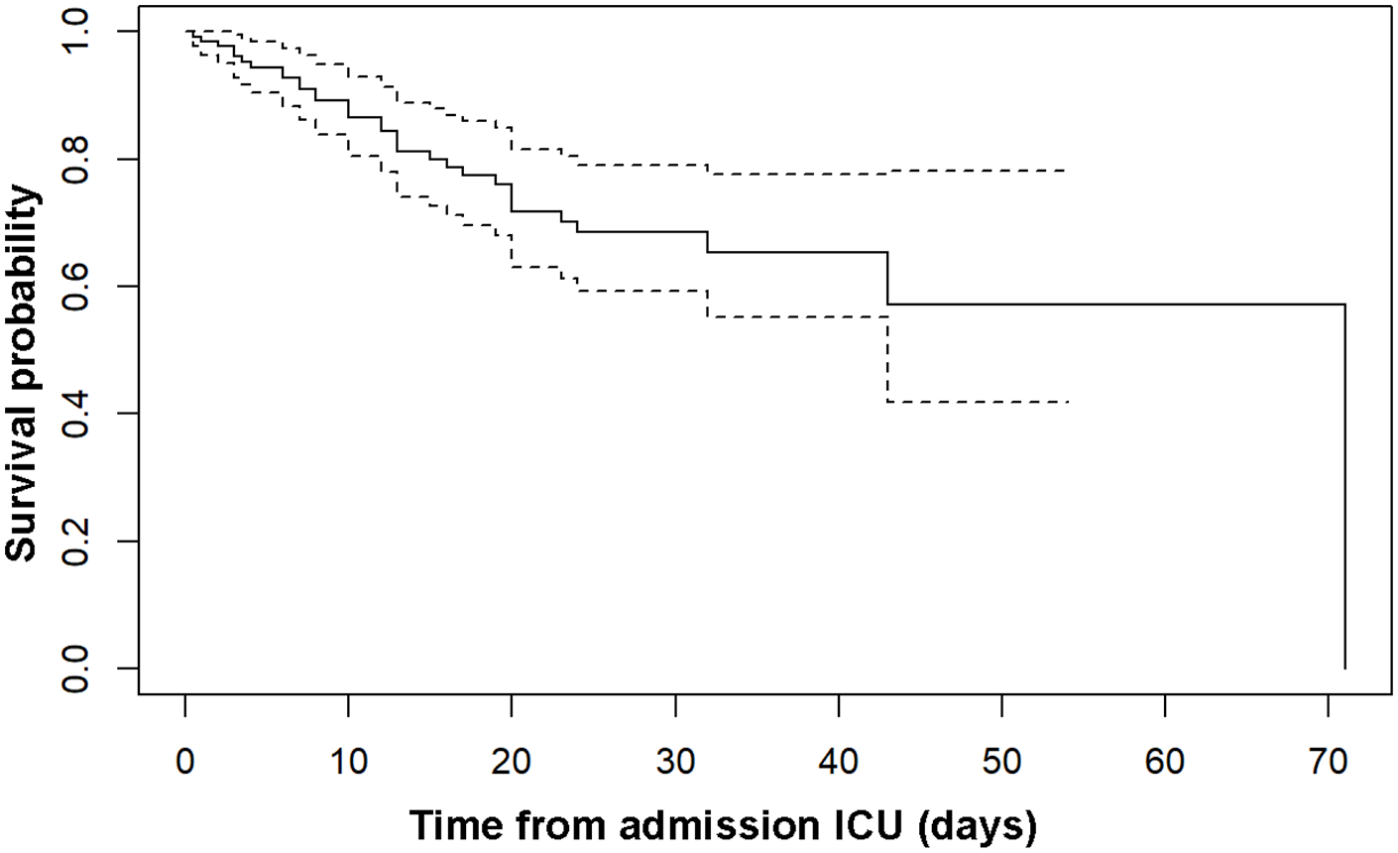
Kaplan-Meier curve of survival. Abbreviation: ICU, intensive care unit.

Baseline characteristics are presented in Table 1. There was no significant difference between survivors and non-survivors in sex, age, BMI, or any of the comorbidities. The Kaplan-Meier curve for survival is shown in Figure 1.

**Table 1.** Baseline characteristics.

Patient characteristics		All patients	Survivors	Non-survivors	*P*-value
		n = 107	n = 81	n = 26	
Gender: male	n (%)	79 (73.8)	57 (70.4)	22 (84.6)	.238
Age	Median (IQR)	64 (16)	61 (16)	68 (18.5)	.067
BMI	Mean (*SD*)	29.1 (7.0)	29.1 (6.5)	29.3 (8.3)	.866
Comorbidity: pulmonary disease	n (%)	20 (18.6)	16 (19.8)	4 (15.4)	.835
Comorbidity: cardiovascular disease	n (%)	46 (43.0)	33 (40.7)	13 (50.0)	.407
Comorbidity: diabetes mellitus	n (%)	29 (27.1)	24 (29.6)	5 (19.2)	.432
Comorbidity: malignancy	n (%)	8 (7.5)	5 (6.2)	3 (11.5)	.634
Comorbidity: renal disease	n (%)	3 (2.8)	1 (1.2)	2 (7.7)	.292

Abbreviation: BMI, body mass index.

In a Cox regression model including age and gender, the HR of age for in-hospital mortality was 1.036 (1.001-1.072) and that of female sex was 0.344 (0.105-1.131). We saw that the effect of BMI was weak and removed it from subsequent analysis.

There were a total of 1336 PCT measurements in 92 patients, 811 suPAR measurements in 92 patients, and 1286 IL-6 measurements in 91 patients.

The HRs of PCT, IL-6, suPAR, and CRP are shown in Table 2. Adjusted for sex and age, a doubling in the next day in either levels of PCT, IL-6, and suPAR was significantly predictive of in-hospital mortality with and an HR of 1.523 (1.012-6.540), 75.25 (1.116-6247), and 24.45 (1.696-1057) respectively.

**Table 2.** Hazard ratios on mortality of different biomarkers.

	HR	2.5%	97.5%	*P*-value
Procalcitonin				
Age	1.032	0.9344	1.129	.4501
Gender: female	0.5344	0.08998	3.521	.4165
2 Fold increase	1.523	1.012	6.54	.03253
20% increase	1.117	1.003	1.639	.03253
suPAR				
Age	1.021	0.9432	1.152	.689
Gender: female	0.3688	0.01645	2.732	.344
2 Fold increase	24.46	1.696	1057	.007067
20% increase	2.319	1.149	6.243	.007067
IL-6				
Age	1.037	0.9761	1.115	.2858
Gender: female	0.366	0.05727	1.537	.2111
2 Fold increase	75.24	1.116	6247	.0444
20% increase	3.116	1.029	9.963	.0444
CRP				
Age	1.049	0.9879	1.123	.1295
Gender: female	0.5067	0.1095	1.756	.335
2 Fold increase	14.55	0.21	1518	.2305
20% increase	2.022	0.6633	6.867	.2305

Abbreviations: CRP, C-reactive protein; HR, hazard ratio; IL-6, interleukin-6; suPAR, soluble urokinase-type plasminogen activator receptor.

With a 20% increase in biomarker value, the HR of PCT, IL-6, and suPAR were 1.117 (1.03-1.639), 3.116 (1.029-9.963), and 2.319 (1.149-6.243) respectively. A doubling of CRP levels was no significant predictor of in-hospital mortality with an HR of 14.55 (0.21-1518) and neither was a 20% increase of CRP with a HR of 2.022 (0.663-6.867).

## Discussion

In this exploratory study we investigated the predictive value of the trend in repeated measurements of different biomarkers of disease severity and inflammation for ICU mortality in COVID-19 patients. We found that when IL-6, suPAR, or PCT double or rise with 20% in a subsequent day that this is predictive of in-hospital mortality. These findings confirm that these biomarkers are predictors of disease severity, and add that a rising trend in these biomarker values predicts mortality in the ICU in COVID-19 patients.

In clinical practice, trends and changes in biomarkers are used daily to monitor a patient’s status and to evaluate if a disease of the patient is progressing or resolving.^14^ However, the actual effect or prognostic value of a certain rise in biomarkers is often unknown and rarely investigated in clinical studies. Our study shows how joint models can be translated to data that can used in daily clinical practice. We showed that a trend, such as a doubling of 20% increase, in biomarkers predicts mortality, which may help physicians identifying patients that require more intensive treatment, especially when ICU capacity is stressed due to a pandemic. Furthermore, clinical deterioration may be detected before vital parameters further worsen when looking at the daily changes in these biomarkers. When patients at risk of mortality are detected early, more intensive diagnostic work-up or treatment could be initiated, potentially averting further deterioration. To evaluate if such approach would benefit the clinical outcome, validation in an interventional study is required.

The analysis of daily repeated measurements to investigate the relation of a trend in time with a survival outcome require appropriate statistical methods to correctly interpret the data. In contrast to a cross sectional design or single point measurement, a regular Cox or logistic regression analysis cannot be used. Joint models allow the simultaneous modeling of a longitudinal outcome such as a daily biomarker measurement in the ICU, and a time-to-event outcome, which was ICU mortality in this study.^15^

We chose to investigate suPAR, IL-6, and PCT because these biomarkers are derived from different inflammatory pathways. They have previously been investigated in COVID-19 patient as single measurements.^6^ SuPAR is a general marker of disease severity and has shown to be elevated in different kind of infections.^16^ SuPAR at admission is a predictor of severe complications.^17^ However, no studies have been done investigating the predictive value of suPAR in ICU patients with COVID-19.Although we found that a rise in suPAR is predictive of mortality, translating these results to clinical practice may be challenging. SuPAR was already elevated in all patients at admission. The detection limit of suPAR was 25 ng/mL, resulting in 29% of the measurements above the detection limit. The range of detection of suPAR is therefore too narrow for severely ill patients, such as COVID-19 patients. The role of IL-6 in COVID-19 patients has been investigated extensively, because selective inhibition of IL-6 may improve survival.^18^ In a study by Gorham et al the use of repeated measurements of IL-6 was investigated. Even though this study used daily measurements of IL-6, the authors only used the changes between predetermined time points and admission. The strength of our study is that we showed that a rise in biomarker level in a following day, no matter which admission day, predicts mortality. PCT has previously been investigated as bacterial marker. Currently, its main role in the ICU is to aid the clinical decision to start or stop antibiotic treatment.^19^ In COVID-19 patients, PCT may aid in identifying patients with bacterial coinfections.^7^ Several studies showed that PCT is also a marker of disease severity.^8,9,20^ Our findings support that PCT is a biomarker of disease severity, although we did not correct for bacterial coinfections in our patients.

Different other biomarkers have been identified as predictor of mortality in COVID-19 patients when measured at hospital admission.^21,22^ However, comparing these findings to our study is challenging, because the predictive value of a biomarker at admission may not be the same as the predictive value of the trend of the same biomarker during admission, as illustrated by our finding that the trend of CRP is no predictor of mortality. An increase in CRP level was not significantly associated with mortality in our study. This is in contrast to several studies that showed that elevated CRP levels at ICU admission is predictive of mortality in COVID-19 patients.^23,24^ We hypothesize that the up- and down-regulating factors influencing the daily trend of CRP levels are too diverse, in severely ill COVID-19 patients in the ICU, resulting in a trend that is not significantly predictive of mortality.

The research field in prediction models is shifting toward the use of more advanced technological models, such as machine learning for processing large amount of data.^25^ Using repeated measurements allows for more personalized medicine.^26^ Certain biomarkers, such as suPAR, can be elevated in chronic condition like kidney diseases and malignancies.^27,28^ Therefore, when the absolute value is already elevated at admission, it is more informative to look at relative changes in time, which contributes to more personalized medicine. The use of repeated measurements to predict certain outcomes in the ICU is in itself a well-known concept. A study by Lu et al^29^ used linear mixed-effects sub-models in COVID-19 patients to predict mortality using repeated SpO_2_/FiO_2_ ratios and showed that unit decrease in the ratio corresponded to 1.82-fold increase in mortality risk. Our study shows that the method of joint models is feasible in the ICU where laboratory data are collected daily and vital parameters are continuously monitored and recorded.^30^ Future studies should incorporate these continuously measured parameters in combination with biomarkers, which could result in a more accurate mortality prediction when more predictors are used.

## Limitations

This study has several limitations. Because it is an explorative and retrospective study to investigate the concept of using repeated measurements, the study used a convenience sample of all COVID-19 patients who were admitted to the ICU in the spring of 2020. The mortality rate was relatively low with 26 patients who died. Therefore, the findings of this study are at risk of overfitting. Furthermore, due to the small sample size, we could not develop a more precise prediction model that would also correct for comorbidities and other possible confounders. Although these findings need to be validated in a larger cohort, they do show that the use of joint models in longitudinal data is a feasible method for the prediction of mortality in ICU patients. Furthermore, the biomarkers that were investigated in this study were prospectively measured and available to the treating physicians. The outcomes of the study may therefore be biased when physicians used these biomarkers for monitoring or clinical decision making.

## Conclusion

Joint models for the analysis of repeated measurements of PCT, suPAR, and IL-6 are a useful method for predicting mortality in COVID-19 patients in the ICU. Patients with an increasing trend of biomarker levels in consecutive days are at increased risk for mortality.
